# 2677. Evaluation of Expedited Partner Therapy Prescribing in an Urban Emergency Department

**DOI:** 10.1093/ofid/ofad500.2288

**Published:** 2023-11-27

**Authors:** Nicholson B Perkins, William Campillo Terrazas, Casey Boyer, Rachel M Kenney, Nancy MacDonald

**Affiliations:** Henry Ford Hospital, Hernando, Mississippi; Henry Ford Hospital, Hernando, Mississippi; Henry Ford Hospital, Hernando, Mississippi; Henry Ford Hospital, Hernando, Mississippi; Henry Ford Hospital, Hernando, Mississippi

## Abstract

**Background:**

Expedited Partner Therapy (EPT) is a useful tool that facilitates prompt treatment of sex partners in patients diagnosed with sexually transmitted infections (STIs). This study describes EPT utilization and documentation practices in an urban Emergency Department (ED) after implementing electronic EPT orders into a medical record system.

**Methods:**

This retrospective, cross-sectional study evaluated EPT prescribing and documentation practices in an urban ED from 1/1/22 – 9/30/22. The study included patients older than 18 years of age with suspected chlamydia, gonorrhea, and/or trichomoniasis who received either a physical EPT prescription or an EPT kit. Collected data included patient characteristics, diagnostic and treatment data, and documentation measures. Descriptive statistics were used to assess the number of STI cases and those who received EPT.

**Results:**

Data was collected on 37 patients who received EPT during their ED visit. The majority were uninsured (43%), and each patient had an average of 1.27 sex partners. HIV and syphilis screening were performed only in 8 (22%) and 7 (19%) of patients seen for a suspected STI. The number of cases for chlamydia (n=228), gonorrhea (n=149), and trichomoniasis (n=282) were disproportional to the total number of EPT orders throughout the 9-month study period. The number of EPT prescriptions was highest after the implementation of electronic sentences (n=27). At discharge, 76% of patients received a medication kit to provide direct treatment access for sex partners of index patients. Electronic dot phrases were used by 81% of providers to meet state documentation requirements. The results of this study demonstrate a significant STI burden with low EPT utilization from providers.

**Conclusion:**

There was an increase of EPT utilization after the implementation of electronic order sentences in the electronic medical record. Screenings for HIV and syphilis are an area for improvement among patients at risk for an STI who are seen in the ED. The integration of electronic EPT methods can help address the growing rates of STIs in the U.S.

**Disclosures:**

**All Authors**: No reported disclosures

